# Chemokine Receptor Expression on Normal Blood CD56^+^ NK-Cells Elucidates Cell Partners That Comigrate during the Innate and Adaptive Immune Responses and Identifies a Transitional NK-Cell Population

**DOI:** 10.1155/2015/839684

**Published:** 2015-10-12

**Authors:** Margarida Lima, Magdalena Leander, Marlene Santos, Ana Helena Santos, Catarina Lau, Maria Luís Queirós, Marta Gonçalves, Sónia Fonseca, João Moura, Maria dos Anjos Teixeira, Alberto Orfao

**Affiliations:** ^1^Laboratory of Cytometry, Service of Hematology, Hospital de Santo António (HSA), Centro Hospitalar do Porto (CHP), Rua D. Manuel II, 4050-345 Porto, Portugal; ^2^Laboratory of Flow Cytometry, Centro de Investigación del Cancer (CIC), Campus Miguel de Unamuno, 37007 Salamanca, Spain

## Abstract

Studies of chemokine receptors (CKR) in natural killer- (NK-) cells have already been published, but only a few gave detailed information on its differential expression on blood NK-cell subsets. We report on the expression of the inflammatory and homeostatic CKR on normal blood CD56^+low^ CD16^+^ and CD56^+high^  CD16^−/+low^ NK-cells. Conventional CD56^+low^ and CD56^+high^ NK-cells present in the normal PB do express CKR for inflammatory cytokines, although with different patterns CD56^+low^ NK-cells are mainly CXCR1/CXCR2^+^ and CXCR3/CCR5^−/+^, whereas mostly CD56^+high^ NK-cells are CXCR1/CXCR2^−^ and CXCR3/CCR5^+^. Both NK-cell subsets have variable CXCR4 expression and are CCR4^−^ and CCR6^−^. The CKR repertoire of the CD56^+low^ NK-cells approaches to that of neutrophils, whereas the CKR repertoire of the CD56^+high^ NK-cells mimics that of Th1^+^ T cells, suggesting that these cells are prepared to migrate into inflamed tissues at different phases of the immune response. In addition, we describe a subpopulation of NK-cells with intermediate levels of CD56 expression, which we named CD56^+int^ NK-cells. These NK-cells are CXCR3/CCR5^+^, they have intermediate levels of expression of CD16, CD62L, CD94, and CD122, and they are CD57^−^ and CD158a^−^. In view of their phenotypic features, we hypothesize that they correspond to a transitional stage, between the well-known CD56^+high^ and CD56^+low^ NK-cells populations.

## 1. Introduction

Natural killer- (NK-) cells were originally identified by their natural ability to kill target cells and are known for a long time as effector cells of the innate immune system, with an important role in controlling several types of tumors and infections [1]. In recent years, NK-cells have also been recognized as regulatory cells, which are able to interact with other cells of the immune system, such as dendritic cells (DC), monocytes/macrophages, and T cells, thereby influencing the innate and adaptive immune responses [2–5]. The role of their interaction with neutrophils in shaping the immune response is also being increasingly documented [6, 7].

The cytotoxic activity of the NK-cells is controlled by the balance between inhibitory and activating receptors, whose ligands are self-Major Histocompatibility Complex (MHC) class I molecules and molecules expressed on stressed, viral infected, and tumor cells. They comprise, among others, the killer cell immunoglobulin-like receptors (KIR), killer cell lectin type receptors (KLR), and natural cytotoxic receptors (NCR) as well as immunoglobulin Fc receptors (FcR) and complement receptors [8–10].

Meanwhile, the immunoregulatory properties of the NK-cells are mediated, not only by cell-to-cell contact, but also by the soluble factors they produce, which enable them to recruit and to activate other immune cells. These include chemokines (CK), such as MIP-1*α* (macrophage inflammatory proteins-1 alpha, CCL3) and MIP-1*β* (CCL4), RANTES (regulated activation, normal T cell expressed and secreted, CCL5), and ATAC (activation-induced, T cell derived, and chemokine-related cytokine, CXCL1). They also comprise cytokines, for example, IFN-*γ* (interferon-gamma) and TNF-*α* (tumor necrosis factor alpha) and growth factors, such as GM-CSF (granulocyte-macrophage colony-stimulating factor) [11, 12].

Using adhesion molecules and chemokine receptors (CKR), NK-cells are able to circulate in the blood and to distribute throughout the body, by homing into secondary lymphoid organs (e.g., lymph nodes), localizing in specific nonlymphoid organs (e.g., liver, placenta), and migrating into acute or chronic inflamed tissues, where they participate in the immune responses [13–16]. In some organs, NK-cells exhibit specific phenotypes and functions [17, 18], for example, promoting decidualization of the endometrium, embryo implantation and placenta development [19, 20], and influencing the hematopoiesis [21, 22].

Two different subsets of mature CD56^+^ NK-cells have been described in humans, based on the levels of CD56 and CD16 expression: CD56^+low^ CD16^+^ and CD56^+high^  CD16^−/+low^ NK-cells from now on designed CD56^+high^ and CD56^+high^, respectively [23, 24]. While the former clearly predominates in the peripheral blood (PB), where they represent around 90% of the circulating CD56^+^ NK-cells, the latter are more represented in secondary lymphoid organs, chronically inflamed tissues and placenta [13–16, 19, 20].

Apart from the different expression of CD16, the low affinity receptor for IgG (Fc*γ*RIIIA) and CD56, the neural cell adhesion molecule (NCAM), the conventional CD56^+^ NK-cell subsets also differ in the expression of other adhesion, homing, and costimulatory molecules as well as on the repertoires of NCR, KIR and KLR, and receptors for cytokines, chemokines, and growth factors [25–29]. In addition, these NK-cell subsets exhibit distinct sialylated forms of CD43 and posttranslational modifications of the P-selectin glycoprotein ligand-1 (PSGL-1) [30, 31].

From the functional point of view, CD56^+low^ NK-cells are essentially cytotoxic, with a greater level of antibody dependent cell mediated cytotoxicity (ADCC) [32], whereas CD56^+high^ NK-cells have a high proliferative response to low doses of interleukin- (IL-) 2 (IL-2) and C-kit ligand [33]. In addition, the latter display a more important immunomodulatory role associated with cytokine production in response to IL-2 and monokines [33]. More recently it became apparent that upon target cell recognition, CD56^+low^ NK-cells are more prominent cytokine and chemokine producers than CD56^+high^ NK-cells [34]. These diverse functional properties would suggest that CD56^+low^ and CD56^+high^ NK-cells could be naturally prepared to act in different sites and at different phases of the immune response.

The exact relationship between these NK-cell subsets still remains unclear. Some studies have shown that bone marrow progenitor cells give rise to CD56^+high^ or CD56^+low^ NK-cells depending on being cultured in the presence of IL-15 alone or in combination with IL-21, respectively [35, 36]. However, more recent data would favor a possible maturation relationship between these NK-cell subsets and suggest that CD56^+low^ NK-cells originate from CD56^+high^ NK-cells [37–42].

Chemokines are small proteins that control a number of biological activities, including cell development, differentiation, tissue distribution, and function [43]. They act by binding chemokine receptors (CKR), a family of seven-transmembrane proteins that are classified by structure according to the number and spacing of conserved cysteines into four major groups given the names CXCR, CCR, CX3CR, and XCR to which four groups of CK correspond: CXCL, CCL, CL, and CX3CL [44]. In addition, CXCL chemokines have been further subclassified into glutamic acid-leucine-arginine tripeptide (ELR) positive or negative, based on the presence or absence of the ELR motif N-terminal to the first cysteine. From a functional point of view, two distinct types of CK have been considered: inflammatory/inducible CK, which are regulated by proinflammatory stimuli and dictate migration to the inflamed tissues and homeostatic/constitutive CK, which are responsible for the homing of the immune cells to the lymphoid organs and tissues. Similarly, two distinct groups of CKR have been described: those that interact mainly with inflammatory/inducible CK and have overlapping specificities and those that are relatively specific for homeostatic/constitutive CK [43, 44].

To the best of our knowledge only a few studies analyzed in detail the CKR repertoire on CD56^+low^ and CD56^+high^ NK-cells and the results obtained were somewhat divergent [45, 46]. For instance, Campbell et al. have found that CD56^+^/CD16^+^ (primarily CD56^+low^) NK-cells uniformly express high levels of CXCR1, CXCR4, and CX3CR1 and low levels of CXCR2 and CXCR3 but no CCR1–6, CCR9, CXCR5, and CXCR6; they also found that CD56^+^/CD16^−^ (primarily CD56^+high^) NK-cells do express CXCR3, CXCR4, CCR5, and very low levels of CX3CR1, but no CXCR1, CXCR2, CXCR5, CCR1–4, 6, and 9 [45]. In contrast, Berahovich et al. observed that NK-cells are CXCR1^+^, CXCR3^+^, and CXCR4^+^ and contain subsets expressing CCR1, CCR4, CCR5, CCR6, CCR9, CXCR5, and CXCR6 [46]; according to their work, with the exception of CCR4, these CKR are expressed at higher percentages by CD56^+high^ NK-cells [46]. Additionally, both authors have found CCR7 to be restricted to CD56^+high^ NK-cells, which has been proved to regulate its selective homing into the lymph nodes (LN) [47, 48], where these cells establish the link between innate and adaptive immunity [47, 48].

We have previously characterized the immunophenotype of blood CD56^+low^ and CD56^+high^ NK-cells [29]. In order to better understand the migration pathways and cell-interactions of these NK-cell subsets and to establish the normal reference patterns for the study of the NK-cell lymphoproliferative disorders, we decided to investigate the expression of a number of CKR on these NK-cell subsets. At some point in our study, we found that blood CD56^+^ NK-cells include a minor population of CXCR3/CCR5^+^ NK-cells whose levels of CD56 expression are intermediate between those observed on CD56^+low^ and CD56^+high^ NK-cells, most of which are CD16^+^. These cells, from now on referred to as CD56^+int^ NK-cells, fail to display CD57 and KIR, and they have intermediate levels of CD62L, CD94, and CD122 expression. Based on the results presented herein and on the published data, we discuss the migration routes of the conventional CD56^+high^ and CD56^+low^ NK-cells and their relevance for the success of the immune response and hypothesize that CD56^+int^ NK-cells probably represent a transitional NK-cell state.

## 2. Material and Methods

### 2.1. Subjects

We first analyzed by flow cytometry the expression of a number of CKR on the CD56^+^ NK-cells in the PB of 15 adult healthy individuals (blood donors), 9 males and 6 females, aged from 19 to 54 years (median age of 38 years). After suspecting the existence of a subpopulation of CD56^+int^ NK-cells, these cells were further characterized using another group of 13 adult healthy individuals (blood donors), 8 males and 5 females, aged from 20 to 64 years (median age of 40 years).

### 2.2. Ethical Statement

This study was approved by the Ethical Committee as part of a research project aimed to characterize the CKR on normal and neoplastic T cells and NK-cells in order to better understand the biology of the T cell and NK-cell lymphoproliferative disorders. All individuals gave informed consent to participate in the study.

### 2.3. Flow Cytometry Studies

Immunophenotyping was performed using a whole blood stain-lyse-and-then-wash direct immunofluorescence technique using FACS lysing solution (Becton Dickinson, San José, CA) (BD) for erythrocyte lysis and cell fixation and four-color stainings with monoclonal antibodies (mAbs) conjugated with fluorescein isothiocyanate (FITC), phycoerythrin (PE), PE-Cyanine 5 (PC5) or peridinin chlorophyll protein (PerCP), and allophycocyanin (APC). These were purchased to BD, Pharmingen (PH; San Diego, CA), Beckman Coulter (BC; Miami, FL), Immunotech (IOT; Marseille, France), and CLB (Amsterdam, Netherlands). Appropriate fluorochrome-conjugated isotype matched mAbs were used as negative controls.

In order to characterize the CKR expression on the conventional CD56^+low^ and CD56^+high^ NK-cell subsets, APC-conjugated anti-CD3 (BD; mouse IgG1,*κ*; clone SK7), PC5-conjugated anti-CD56 (IOT; mouse IgG1,*κ*; clone N901/NKH-1), and FITC-conjugated anti-CD16 (IOT; mouse IgG1,*κ*; clone 3G8) mAbs were used in combination with PE-conjugated mAbs directed against the following CKR (PH): CXCR1 (CD181) (mouse IgG2b,*κ*; clone 5A12), CXCR2 (CD182) (mouse IgG1,*κ*; clone 6C6), CXCR3 (CD183) (mouse IgG1,*κ*; clone 1C6), CCR4 (CD194) (mouse IgG1,*κ*; clone 1G1), CCR5 (CD195) (mouse IgG2a,*κ*; clone 2D7/CCR5), and CCR6 (CD196) (mouse IgG1,*κ*; clone 11A9).

Subsequently, CD56^+int^ NK-cells (which, in most of the normal PB samples, cannot be distinguished from the conventional CD56^+low^ or CD56^+high^ NK-cells using the staining protocol mentioned above) were further characterized using APC-conjugated anti-CD3, PC5-conjugated anti-CD56, PE-conjugated anti-CXCR3 + PE-conjugated anti-CCR5, and one of the following FITC-conjugated mAbs directed against these molecules: anti-CD16 (IOT; mouse IgG1,*κ*; clone 3G8), anti-CD57 (BD; mouse IgM,*κ*; clone HNK-1), anti-CD62L (BD; mouse IgG2a,*κ*; clone SK11), anti-CD94 (PH; mouse IgG1,*κ*; clone HP-3D9), anti-CD122 (CLB; mouse IgG2a,*κ*; clone MIK-b1), and anti-CD158a (BD; mouse IgM,*κ*; clone HP-3E4).

Data acquisition was carried out in a FACSCalibur flow cytometer (BD) equipped with a 15 mW air-cooled 488 nm argon ion laser and a 625 nm neon diode laser, using the CellQUEST software (BD). Information on a minimum of 2 × 10^5^ events was acquired and stored as FCS 2.0 data files for each staining. For data analysis the Paint-a-Gate PRO (BD) and the Infinicyt (Cytognos, Salamanca, Spain) software programs were used.

Using the first staining protocol, NK-cells were first gated based on their CD3^−^/CD56^+^ phenotype; then, the conventional CD56^+low^ and CD56^+high^ NK-cell subsets were selected based on their levels of CD56 expression and on their differential positivity for CD16 and separately analyzed for the expression of CXCR1, CXCR2, CXCR3, CCR4, CCR5, and CCR6. Using the second staining protocol, in which the anti-CXCR3 and CCR5 mAbs used have the same fluorochrome, we were able to distinguish three populations of CD56^+^ NK-cells: CD56^+low^ CXCR3/CCR5^−^, CD56^+int^ CXCR3/CR5^+^, and CD56^+high^ CXCR3/CR5^+^. These were separately analyzed for the expression of CD16, CD56, CD57, CD62L, CD94, CD158a, and CD122.

The percentage of positive cells, the mean fluorescence intensity (MFI, expressed as arbitrary relative linear units scaled from 0 to 10,000), and the coefficient of variation of the MFI (CV, expressed as percentage) were recorded for each molecule tested.

### 2.4. Statistical Analysis

For all quantitative variables under study, mean, standard deviation, median, and range values were calculated. The statistical significance of the differences observed between groups was evaluated using the Mann-Whitney *U*-test (SPSS 10.0, SPSS, Chicago, IL, USA). *P* values less than 0.05 were considered to be associated with statistical significance.

## 3. Results

### 3.1. Chemokine Receptors on Blood CD56^+low^ and CD56^+high^ NK-Cells

**Figure 1:**
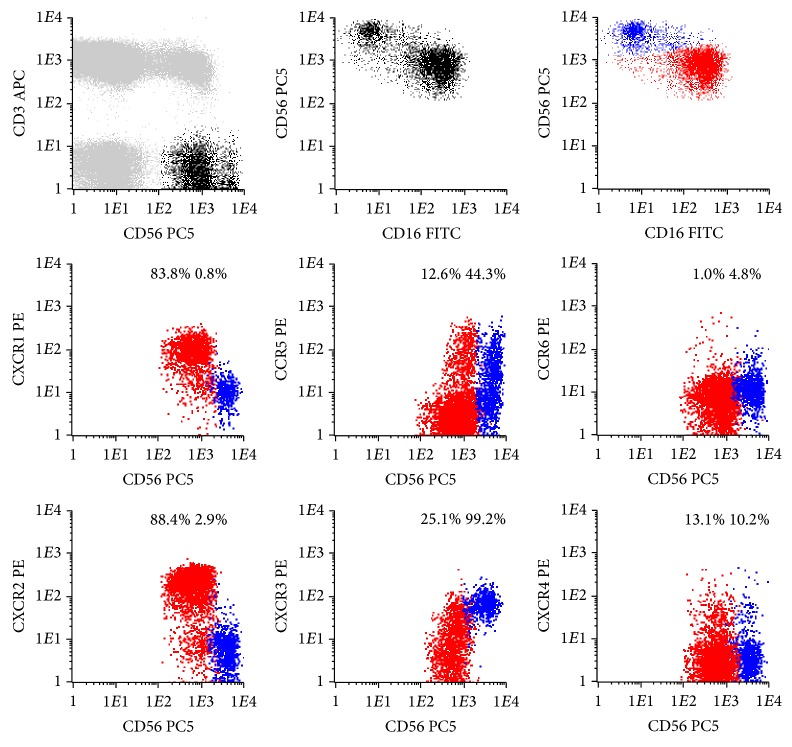
Representative dot plots illustrating the expression of different chemokine receptors (CKR) on the conventional CD56^+low^ (red dots) and CD56^+high^ (blue dots) NK-cell subsets present in the normal peripheral blood (PB). In order to obtain the dot plots showed in this figure, PB cells were stained with APC-conjugated anti-CD3, PC5-conjugated anti-CD56, PE-conjugated anti-CKR, and FITC-conjugated anti-CD16 monoclonal antibodies. Dot plots in the first row illustrate the strategy of gating. Using the CD3/CD56 dot plot, CD56^+^ NK-cells were first identified based on their CD3^−^/CD56^+^ phenotype (black dots), comparatively to T (CD3^+^) and B (CD3^−^CD56^−^) cells (gray dots). Then, after gating for CD56^+^ NK-cells (first CD56/CD16 dot plot), the CD56^+low^ (red dots) and CD56^+high^ (blue dots) NK-cell populations were identified based on their typical patterns of CD56 and CD16 expression (second CD56/CD16 dot plot). Finally, these NK-cell populations were analyzed for the expression of the CKR (CKR/CD56 dot plots). The numbers above the CD56^+low^ and CD56^+high^ NK-cells inside the CKR/CD56 dot plots indicate the percentage of cells staining positively for the correspondent CKR and were obtained after gating separately for each NK-cell population (CKR/CD56 dot plots gated for CD56^+low^ and CD56^+high^ NK-cells are not shown, for simplicity).

Conventional CD56^+low^ and CD56^+high^ NK-cells present in the normal PB have different CKR repertoires (Figure 1 and Table 1).

#### 3.1.1. Chemokine Receptors on Conventional CD56^+low^ NK-Cells

Most CD56^+low^ NK-cells are CXCR1/CXCR2^+^; that is, the majority expresses high levels of CXCR1 (93.0 ± 4.5%) and CXCR2 (91.9 ± 3.4%), whose ligands are CXCL8 (IL-8) and other ELR motif containing chemokines involved in inflammation and angiogenesis [49] (Table 1 and Figure 1). In addition, these NK-cells are CXCR3/CCR5^−/+^, which means that a variable proportion of them have low levels of CXCR3 and/or CCR5 (15.6 ± 11.1% and 13.3 ± 8.8%, resp.) (Table 1 and Figure 1). CXCR3 binds IFN-*γ* inducible cytokines, such as CXCL9 (monokine induced by gamma-interferon, MIG), CXCL10 (interferon-induced protein of 10 kD, IP-10), and CXCL11 (interferon-inducible T cell alpha chemoattractant, I-TAC) and mediates Ca^++^ mobilization and chemotaxis [50–52]. On the other hand, CCR5 has affinity to CCL3 (MIP-1*α*), CCL4 (MIP-1*β*), CCL5 (RANTES), and CCL8 (monocyte chemotactic protein-2, MCP-2) [53, 54].

Concerning the expression of constitutive/homeostatic CKR and the fraction of CD56^+low^ NK-cells that expresses CXCR4, a CKR present on most hematopoietic cell types that binds to CXCL12 (stromal cell derived factor type 1, SDF-1) [55, 56] and has been shown to play a pivotal role in hematopoiesis [57] is variable (21.8 ± 8.7%) (Table 1 and Figure 1). In contrast, CCR4 is expressed in only a very small percentage of the CD56^+low^ NK-cells (0.8 ± 0.4%) (Table 1 and Figure 1). This CKR has been reported to be a marker for T helper 2 (Th2) lymphocytes [58] and promotes homing of memory T cells to inflamed skin [59] by means of interaction with CCL17 (thymus and activation-regulated chemokine, TARC) and CCL22 (macrophage-derived chemokine, MDC) [60, 61]. Similar results were obtained for CCR6, which is expressed in only 0.6 ± 0.4% of the CD56^+low^ NK-cells (Table 1). This CKR mediates responsiveness of memory T cells to CCL3 (MIP-1*α*) [62] and CCL20 (liver- and activation-regulated chemokine, LARC) [63] and has also been implicated in the homing of Langerhans' cells to the epidermis [64].

#### 3.1.2. Chemokine Receptors on Conventional CD56^+high^ NK-Cells

In contrast to CD56^+low^ NK-cells, the majority of the CD56^+high^ NK-cells are CXCR1/CXCR2^−^ and CXCR3^+^; that is, most of CD56^+high^ NK-cells express high levels of CXCR3 (96.9 ± 2.5%) whereas only a few are CXCR1^+^ (4.0 ± 3.6%) or CXCR2^+^ (2.0 ± 1.4%), and a large fraction of them (50.0 ± 15.3%) is CCR5^+^ (Table 1 and Figure 1).

Constitutive/homeostatic CKR are also present in CD56^+high^ NK-cells, with a variable fraction of them expressing CXCR4 (11.4 ± 4.6%) and only a few being CCR4^+^ (3.3 ± 2.9%) and CCR6^+^ (0.9 ± 1.2%, resp.) (Table 1 and Figure 1).

### 3.2. Identification of a New CD56^+int^ NK-Cell Population in the Peripheral Blood

**Figure 2:**
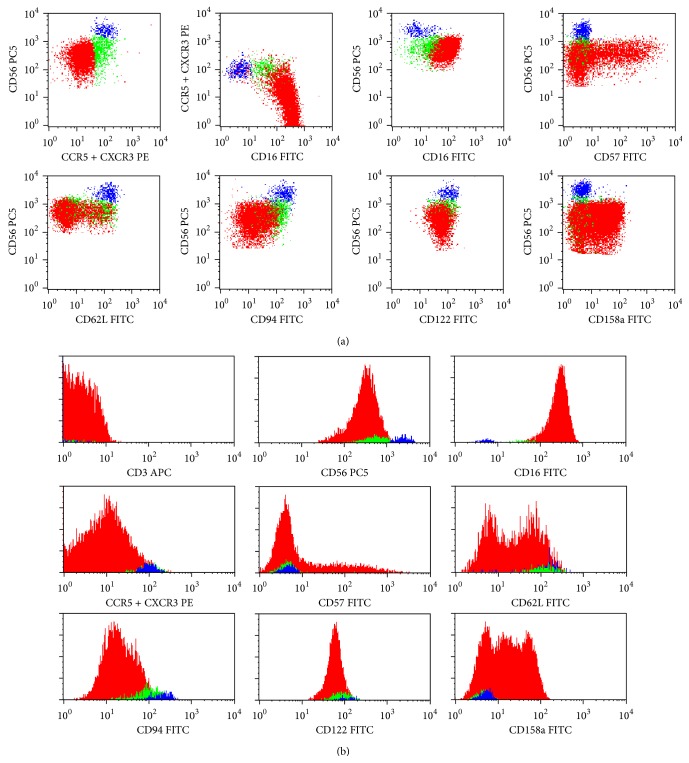
Representative dot plots (a) and histograms (b) illustrating the expression of the CD3, CD16, CD56, CD57, CD62L, CD94, CD122, and CD158a molecules on CD56^+low^ CXCR3/CCR5^−^ (red dots), CD56^+int^ CXCR3/CCR5^+^ (green dots), and CD56^+high^ CXCR3/CCR5^+^ (blue dots) NK-cells in normal peripheral blood (PB). In order to obtain the dot plots showed in this figure, PB cells were stained with APC-conjugated anti-CD3, PC5-conjugated anti-CD56, PE-conjugated anti-CXCR3 + PE-conjugated anti-CCR5, and FITC-conjugated monoclonal antibodies against CD16, CD57, CD62L, CD94, CD122, or CD158a molecules. Total CD56^+^ cells were gated using the strategy illustrated in Figure 1. Then, using the CD56/CCR5 + CXCR3 dot plot (first dot plot), three different CD56^+^ NK-cell populations were identified based on the levels of expression of CD56 and CXCR3/CCR5: CD56^+low^ CCR5/CXCR3^−^ (red dots), CD56^+int^ CCR5/CXCR3^+^ (green dots), and CD56^+high^ CCR5/CXCR3^+^ (blue dots). As it can be seen in the remaining dot plots and histograms, these NK-cell populations differ on the expression of several cell surface molecules. The percentage of cells staining positively for each molecule analyzed, as well as the mean fluorescence intensity of antigen expression and its coefficient of variation, was calculated after gating separately for each NK-cell population and is shown in Table 1 (data is not shown in the figure, for simplicity).

When analyzing the conventional CD56^+^ NK-cell subsets, we observed that the percentage of CD56^+low^ NK-cells staining for CCR5 correlated positively with the percentage of CD56^+low^ NK-cells staining for CXCR3 (*r* = 0.656; *P* = 0.01). In addition, we found that CCR5^+^ and CXCR^+^  CD56^+low^ NK-cells had higher levels of CD56 and lower levels of CD16, as compared to CCR5^−^ (*P* = 0.001 and *P* = 0.05, resp.) and CXCR3^−^ (*P* = 0.003 and *P* = 0.002, resp.) CD56^+low^ counterparts. These observations allow us to investigate if CD56^+low^ cells expressing CCR5 and/or CXCR3 could represent a specific stage in NK-cell differentiation. In accordance, using another staining protocol in which anti-CXCR3 and anti-CCR5 mAbs had the same fluorochrome, we were able to identify three NK-cell populations in the normal PB, based on the expression of CD56, CD16, and the chemokine receptors CXCR3 and/or CCR5 (Figure 2): CD56^+low^ CD16^+^ CCR5/CXCR3^−^ (or simply CD56^+low^), CD56^+int^ CD16^+/−^ CCR5/CXCR3^+^ (or simply CD56^+int^), and CD56^+high^  CD16^−/+low^ CCR5/CXCR3^+^ (or simply CD56^+high^) NK-cells.

In the normal PB, CD56^+low^ NK-cells correspond to the majority (mean of 90 ± 4%) of CD56^+^ NK-cells, whereas the CD56^+int^ and CD56^+high^ NK-cells are minimally represented (mean of 6 ± 4% and 4 ± 2%, resp.) (Table 2).

**Figure 3:**
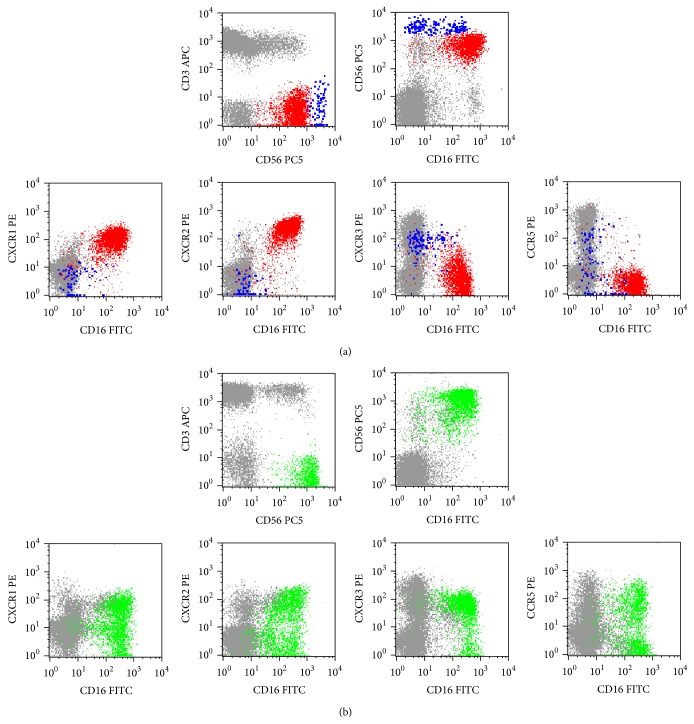
Illustrative dot plots showing the expression of the CXCR1, CXCR2, CXCR3, and CCR5 chemokine receptors (CKR) in normal peripheral blood (PB) (a), where only CD56^+low^ (red dots) and CD56^+high^ (blue dots) are observed, and in the PB of a patient with a chronic lymphoproliferative disorder of NK-cells (CLPD-NK) (b), exhibiting a transitional CD56^+int^ phenotype (green dots); other lymphocytes are shown in gray. In order to obtain the dot plots showed in this figure, the PB cells were stained with APC-conjugated anti-CD3, PC5-conjugated anti-CD56, PE-conjugated anti-CKR (CXCR1, CXCR2, CXCR3, or CCR5), and FITC-conjugated anti-CD16 monoclonal antibodies. As shown in (a), in the normal PB most CD56^+low^ NK-cells are CXCR1^+^ and CXCR2^+^, whereas only a very small fraction of cells stains positively for CXCR3 and/or CCR5; in contrast, most CD56^+high^ NK-cells are CXCR3^+^ whereas CCR5 is expressed in only a fraction and CXCR1 and CXCR2 are virtually negative. As it can be seen in (b), the expanded NK-cells from this patient, which have relatively high levels of CD56 expression, are CD16^+^ and they express the CXCR1, CXCR2, CXCR3, and CCR5 molecules in a considerable fraction of cells.

Despite representing a minor NK-cell population in most normal PB samples, CD56^+int^ NK-cells are largely expanded in some patients with chronic lymphoproliferative disorders of NK-cells (CLPD-NK) (Figure 3).

No differences were observed between these three NK-cell subsets concerning both the cell size and complexity, as evaluated by the forward (FSC) and side light scatter (SSC), respectively, except for a slightly larger size of CD56^+high^ NK-cells (Table 2). Nonetheless, statistically significant differences were found concerning the expression of CD56 and CD16 (Figure 2 and Table 3) as well as of the other adhesion molecules and homing, cytokine, and killer cell receptors analyzed (Figure 2 and Table 4).

#### 3.2.1. Phenotypic Characterization of Blood CD56^+int^ NK-Cells

CD56^+int^ NK-cells do express CD56 at levels that are intermediate between those observed on CD56^+low^ and CD56^+high^ NK-cells (MFI of 615 ± 149, 466 ± 108, and 2926 ± 578, resp.) (Figure 2 and Table 3). They also have intermediate percentages of CD16^+^ cells (64.6 ± 23.6%), as compared to CD56^+low^ and CD56^+high^ NK-cells (99.9 ± 0.1% and 28.7 ± 9.9%, resp.). In addition, the levels of CD16 expression (MFI of 84 ± 55) were in between those observed on CD56^+low^ and CD56^+high^ NK-cells (MFI of 226 ± 107 and 47 ± 18, resp.).

These three CD56^+^ NK-cell subsets also differ in the expression of other molecules (Figure 2 and Table 4).

Concerning the KLR, only a fraction of CD56^+low^ (47.8 ± 13.7%) expresses dimly CD94, whereas nearly all CD56^+int^ (91.4 ± 6.0%) and CD56^+high^ (98.3 ± 1.5%) are CD94^+^ (Figure 2 and Table 4). Curiously, the levels of CD94 expression on CD56^+int^ NK-cells are in between those observed on CD56^+low^ and CD56^+high^ NK-cells (MFI of 129 ± 34, 71 ± 18, and 228 ± 34, resp.).

With respect to the expression of KIR, an opposite pattern is observed. Indeed, a variable fraction of CD56^+low^ NK-cells is CD158a^+^ (38.9 ± 30.0%), in contrast to that found in CD56^+int^ and CD56^+high^ NK-cells, which are basically CD158a^−^ (mean percentage of CD158a^−^ cells of 9.9 ± 9.0% and 4.1 ± 4.9%, resp.) (Figure 2 and Table 4).

Regarding cell adhesion molecules, the percentage of CD62L^+^ cells is significantly lower among CD56^+low^ (35.4 ± 20.4%), as compared to CD56^+high^ NK-cells (97.3 ± 2.4%), intermediate values being observed in the CD56^+int^ NK-cells (77.3 ± 19.0%). Similar results were obtained for the levels of CD62L expression (MFI of 49 ± 9, 119 ± 21, and 139 ± 30, resp.). In addition, a large fraction of CD56^+low^ NK-cells (66.3 ± 15.6%) expresses variably and heterogeneously the CD57 oligosaccharide, whereas most CD56^+int^ NK-cells fail to express this molecule and CD56^+high^ NK-cells are virtually CD57^−^ (mean % of CD57^+^ cells of 15.3 ± 13.7% and 1.3 ± 1.4%, resp.). Once again, the levels of CD57 expression on CD56^+int^ NK-cells (MFI of 355 ± 166) were in between those observed on CD56^+low^ (MFI of 700 ± 436) and CD56^+high^ NK-cells (MFI of 165 ± 219).

The low affinity receptor for IL-2 and CD122, which is present in virtually all NK-cells, also exhibit intermediate levels on CD56^+int^ cells (MFI of 77 ± 20), as compared to CD56^+low^ (MFI of 46 ± 11) and to CD56^+high^ (MFI of 117 ± 32) NK-cells (Figure 2 and Table 4).

## 4. Discussion

In the present study we show that CD56^+low^ and CD56^+high^ NK-cells that circulate in the normal blood have typical and quite different patterns of expression of receptors for inflammatory chemokines. At the same time, we identify and describe a subpopulation of CD56^+int^ NK-cells that could represent a transitional stage in between the conventional NK-cell subsets referred to above, based on their intermediate levels of CD56 and CD16 expression and on their patterns of chemokine (CXCR3, CCR5), cytokine (CD122), and killer cell (CD94, CD158a) receptors and adhesion molecules (CD62L, CD57).

**Figure 4:**
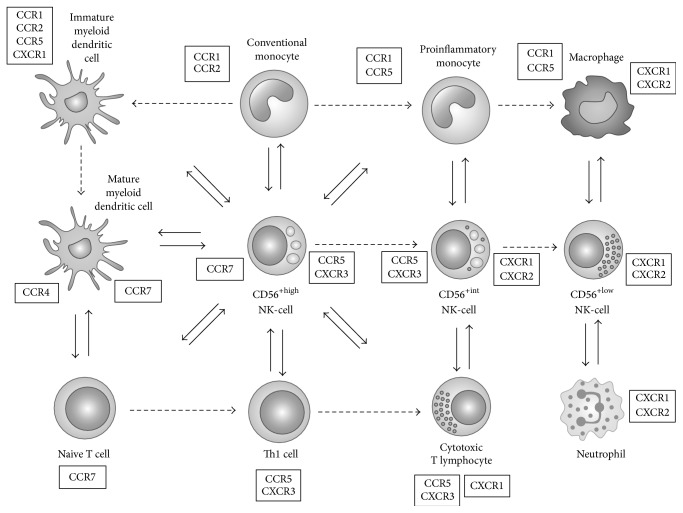
Diagram illustrating the complex relationship established between NK-cells and the other cells of the innate-dendritic cells (DC), monocytes, macrophages, neutrophils, and adaptive (T cells) immune system, whose homing to lymphoid organs and recruitment to inflamed tissues are mediated by the interaction of homeostatic chemokines constitutively expressed on locally resident cells and inflammatory chemokines, with the correspondent chemokine receptors. CCR7 expression on CD56^+high^ NK-cells, mature DC, and naïve T cells allows these cells to migrate into the lymph nodes, in response to CCL19 (ELC) and CCL21 (SLC) produced locally. CXCR3/CCR5 expression on CD56^+high^ NK-cells permits these cells to migrate into inflamed tissues, together with CCR5^+^ proinflammatory monocytes, CCR5/CXCR3^+^ Th1 cells, and CCR5/CXCR3^+^ cytotoxic T lymphocytes (CTL). CXCR1/CXCR2 expression on CD56^+low^ NK-cells, neutrophils, and CTL permits these cells to migrate into inflamed tissues in response to CXCL8 (IL-8), where they interact together and with activated macrophages. CD56^+int^ NK-cells are transitional NK-cells, whose properties are intermediate between those of CD56^+high^ and CD56^+low^ NK-cells. Dashed arrows indicate the routes of differentiation. Full arrows indicate the cross-talk between cells mediated by cytokines and chemokines. CCR7 ligands: CCL-19 (ELC) and CCL21 (SLC); CCR5 ligands: CCL3 (MIP-1*α*), CCL4 (MIP-1*β*), CCL5 (RANTES), and CCL8 (MCP-2); CXCR3 ligands: CXCL9 (MIG), CXCL10 (IP-10), and CXCL11 (I-TAC); CXCL1 ligands: CXCL-8 (IL-8); CXCL2 ligands: CXCL-8 (IL-8) and other ELR motif containing CXCL chemokine; CXCR4 ligand: CXCL12 (SDF-1); CCR1 ligands: CCL3 (MIP-1*α*), CCL5 (RANTES), MCP-2, and MCP-3; CCR2 ligands: CCL2 (MCP-1), CCL8 (MCP-2), CCL7 (MCP-3), and CCL13 (MCP-4).

Differences on the CKR repertoires make the NK-cell subsets naturally able to circulate in the blood, to home into secondary lymphoid organs, or to migrate into inflamed tissues, in different circumstances and with different partners (Figure 4), in response to constitutive and inflammatory chemokines (Table 5).

In accordance, the majority of CD56^+high^ NK-cells are CXCR3/CCR5^+^, a pattern of CKR expression that is typically observed in Th1 cells [65], while CD56^+low^ NK-cells do express CXCR1 and CXCR2, the only CKR specific for the ELR^+^ CXCL chemokines involved in inflammation, thus mimicking neutrophils [66, 67]. In addition, both NK-cell subsets have variable levels of CXCR4 and virtually no CCR4 and CCR6 expression.

CD56^+high^ NK-cells and Th1 cells, the primary cell populations responsible for IL-2, IFN-*γ*, and TNF-*α* production in response to IL-2 or certain monokines, such as IL-12 and IL-15, are attracted together to chronically inflamed tissues in response to CCR5 (MIP-1*α*, MIP-1*β*, RANTES, and MCP-2) and CXCR3 (MIG, IP-10, and I-TAC) chemokine ligands, where they orchestrate the adaptive immune response. Some of these CK, such as RANTES and MIP-1*α*, also attract proinflammatory CD14^+low^ CD16^+^ monocytes, by acting as ligands for CCR1 and CCR4, as well as for CCR5 [68].

In agreement, CD56^+high^ NK-cells accumulate within Th1-type chronic inflammatory lesions in a wide variety of pathological conditions such as rheumatoid arthritis [69], psoriasis [70], sarcoidosis [71], and allograft rejection [72] as well as in sites of intracellular bacterial infections [73], chronic viral infections [74], and tumors [75]. Inside the inflamed tissues and imbibed in the appropriate monokine environment, CD56^+high^ NK-cells are able to engage with monocytes in a reciprocal fashion [76], thereby amplifying the inflammatory response and having important antitumor and antiviral effects. In the LN, they can induce the maturation of DC via IFN-*γ* and TNF-*α* release and/or cell-cell contact-dependent mechanisms [2, 3], in that way shaping the subsequent immune response. Moreover, activated NK-cells can kill immature myeloid DC, which have insufficient amounts of MHC molecules to activate T cells properly [2, 3]. In addition, CD56^+high^ NK-cells also predominate in placenta [77], where they are involved in maternal-fetal tolerance [78, 79].

In contrast, CD56^+low^ NK-cells, which are essentially cytotoxic, and neutrophils, which are phagocytic cells by excellence, predominate in the PB and are equipped with the CXCR1 and CXCR2 chemokine receptors, making them able to comigrate into sites of acute inflammation in response to IL-8 and other ELR motif containing chemokines and to participate in the earliest phase of the innate immune response. As for the neutrophils, migration of CD56^+low^ NK-cells to inflamed tissues also depends on the interaction of different forms of PSGL-1 expressed on their membrane with the selectin molecules expressed on endothelial cells [31]. Curiously, cytotoxic T lymphocytes (CTL) have also been reported to express CXCR1 [80] and PSGL-1 [81].

Normally, both neutrophils, which are able to neutralize efficiently the extracellular pathogens, after opsonization by antibodies, using Fc receptors for IgG and IgA and CD56^+low^ NK-cells, which mediate antibody dependent cell cytotoxicity via Fc*γ*RIIIa (CD16), circulate in the blood. In that sense, it can be hypothesized that these cells are candidates to establish a bridge between the innate immune response and the antibody mediated adaptive immune response. Evidence is being accumulated in the last years for a cross-talk between neutrophils and NK-cells [6, 7]. For instance, NK-cells promote neutrophil recruitment to the inflamed tissues and several NK-cell derived cytokines and growth factors, such as GM-CSF, IFN-*γ*, and TNF-*α*, act by enhancing neutrophil survival and by modulating cell surface expression of complement and Fc receptors in neutrophils [82–84]. On the other hand, neutrophils can stimulate the production of IFN-*γ* by NK-cells [84].

The other anatomical sites in which CD56^+low^ NK-cells and neutrophils might be concomitantly present to modulate each other's activity and its contribution to disease are not completely elucidated. Normal liver contains mainly CD56^+low^ NK-cells, but these cells are different from the CD56^+low^ NK-cells that circulate in the blood [85]. In addition, CD56^+low^ NK-cells also infiltrate the liver of patients with primary biliary cirrhosis, an antibody mediated autoimmune disease, following the CXCR1/IL-8 axis [85]; curiously, hepatic infiltration by neutrophils is also found in these patients [86]. Moreover, CD56^+low^ NK-cells and neutrophils colocalize in the skin of patients with Sweet's syndrome, an acute febrile neutrophilic dermatosis that can follow viral infections, autoimmune diseases, and hematologic malignancies [84].

Also of note, in this study we confirm previous observations about the lack of expression on both CD56^+low^ and CD56^+high^ NK-cells of other CKR involved in homing to nonlymphoid organs and tissues including CCR4 (skin and lung), CCR6 (intestine and liver), CCR9 (small intestine), and CCR10 (skin) [45, 87]. This suggests that, unlike memory/effector T cells, CD56^+^ NK-cells may not be divided into cutaneous versus mucosal/intestinal-homing compartments, based on CKR expression [58, 88, 89].

Of special interest is also the identification of a new population of NK-cells expressing intermediate levels of CD56 that we designed as CD56^+int^ NK-cells. Similar to CD56^+high^ NK-cells, most of the CD56^+int^ NK-cells are KIR^−^ and CD57^−^; however, the majority of them display the CD16 molecule, a marker of CD56^+low^ NK-cells, and they have intermediate levels of CD62L, CD94, and CD122 expression. Due to the fact that the identification of the NK-cell populations by flow cytometry is usually based only on CD56 and CD16 expression, CD56^+int^ NK-cells are being considered together with CD56^+low^ NK-cells on routine blood analysis.

The fact that CD56^+int^ NK-cells have phenotypic features intermediate between those of conventional CD56^+low^ and CD56^+high^ NK-cells would suggest that they could represent a transitional NK-cell maturation stage.

In line with this hypothesis, evidence for the existence of transitional NK-cell populations with phenotypic features similar to those of the CD56^+int^ NK-cells described herein has also been provided in other studies [90, 91]. In accordance, Yu et al. described a CD56^+low^  CD94^+high^ NK-cell subset expressing CD2, CD62L, CD56, KIR, granzymes, and perforin, producing IFN-*γ* in response to monokines, and exhibiting CD94-mediated redirected killing at levels intermediate between those observed in CD56^+low^  CD94^+low^ and CD56^+high^  CD94^+high^ NK-cells [90]. In addition, Juelke el al. reported on a CD56^+low^ CD62L^+^ NK-cell subset with the ability to produce IFN-*γ* and the capacity to kill [91]. Finally, when studying the differentiation of CD56^+high^ CD94/NKG2A^+^ into CD56^+low^ CD94/NKG2A^−^ NK-cells, Béziat et al. found a transitional CD56^+low^ CD94/NKG2A^+^ NK-cell subset, expressing intermediate levels of CD62L, granzyme-K, CD27, and CD57, among other molecules [92]. Given the immunophenotypic similarities, the CD56^+int^ NK-cell population described herein, which comprises less than 10% of the CD56^+^ NK-cells in the PB from normal healthy individuals, probably corresponds to a subpopulation of the CD56^+low^  CD94^+high^ NK-cell subset described by Yu et al., which accounts for half of the circulating CD56^+^ NK-cells [90].

Another potential interest of identifying normal NK-cells with intermediate phenotypic features relies on data interpretation in clinical settings. For instance, overrepresentation of CD56^+int^ NK-cells in the PB from patients with NK-cell lymphocytosis may erroneously be interpreted as phenotypically aberrant (and thus potentially neoplastic) NK-cells. Thus, the knowledge about the immunophenotype of the NK-cell populations that circulate in normal PB, as well as in the PB from patients with inflammatory and infectious conditions [93, 94], is essential to a better understanding of the phenotypic heterogeneity of the expanded NK-cell populations observed in patients with CLPD-NK [95], thereby contributing to distinguishing nonclonal from clonal NK-cell proliferations and reactive from neoplastic conditions [96, 97].

## 5. Conclusions

Differences in the CKR expression on CD56^+low^ and CD56^+high^ NK-cells may determine their ability to be recruited into inflamed tissues and colocalize with other cells at sites of inflammation, which is crucial for the success of the immune response. In addition, the phenotypic heterogeneity of the conventional CD56^+low^ and CD56^+high^ NK-cells may be largely due to the presence of transitional NK-cell populations, which may be preferentially expanded in some pathological conditions.

Further investigations in this area will help to better understand the terminal differentiation of the NK-cells and the maturation relationship between the NK-cell subsets, their circulation through the body, and their participation in the immune response. In addition, they will give an important contribution to establish phenotypic criteria to differentiate reactive and neoplastic NK-cell proliferations, as well as to better identify the normal cell counterparts from which the neoplastic NK-cells originate.

## Figures and Tables

**Table 1:** Chemokine receptor expression on the well-known CD56^+low^ and CD56^+high^ NK-cells observed in blood, as identified based only on the levels of CD56 and CD16 expression.

		CD56^+low^ NK-cells		CD56^+high^ NK-cells		*P* values
CXCR1	% (+) cells	93.0 ± 4.5	(85.3–99.6)	4.0 ± 3.6	(0.0–12.1)	<0.001
	MFI	100.1 ± 19.4	(79.3–140.9)	7.0 ± 3.1	(2.0–14.5)	<0.001
	CV	67.1 ± 23.6	(51.5–145.3)	156.8 ± 76.2	(75.3–329.7)	<0.001

CXCR2	% (+) cells	91.9 ± 3.4	(86.1–97.0)	2.0 ± 1.4	(0.0–4.7)	<0.001
	MFI	165.7 ± 68.6	(66.9–264.1)	3.6 ± 1.5	(2.2–6.8)	<0.001
	CV	64.1 ± 8.7	(48.5–78.6)	218.1 ± 131.4	(82.7–568.9)	<0.001

CXCR3	% (+) cells	15.6 ± 11.1	(4.8–40.1)	97.0 ± 2.5	(92.5–99.6)	<0.001
	MFI	15.5 ± 10.7	(6.5–41.6)	94.5 ± 55.1	(48.7–231.1)	<0.001
	CV	209.8 ± 62.9	(145.1–403.4)	62.7 ± 8.1	(49.6–75.3)	<0.001

CXCR4	% (+) cells	21.8 ± 8.7	(8.4–43.5)	11.4 ± 4.6	(5.5–21.4)	<0.001
	MFI	11.8 ± 3.9	(5.3–20.9)	7.8 ± 3.1	(3.6–14.0)	n.s.
	CV	308.5 ± 89.8	(182.6–492.8)	318.6 ± 98.8	(184.4–581.1)	n.s.

CCR4	% (+) cells	0.8 ± 0.4	(0.2–1.5)	3.3 ± 2.9	(0.3–9.7)	<0.05
	MFI	2.2 ± 0.6	(1.3–3.0)	3.1 ± 1.1	(1.5–4.4)	n.s.
	CV	393.3 ± 195.1	(138.1–677.6)	118.7 ± 107.2	(59.1–417.3)	0.01

CCR5	% (+) cells	13.3 ± 8.8	(2.8–33.2)	50.0 ± 15.3	(24.8–78.5)	<0.001
	MFI	11.1 ± 6.0	(3.5–20.6)	27.8 ± 18.6	(7.7–79.9)	<0.01
	CV	353.5 ± 92.8	(194.8–492.5)	133.9 ± 28.1	(96.7–192.4)	<0.001

CCR6	% (+) cells	0.6 ± 0.4	(0.1–1.3)	0.9 ± 1.2	(0.0–3.0)	n.s.
	MFI	2.3 ± 0.7	(1.5–3.9)	2.1 ± 0.8	(1.4–4.0)	n.s.
	CV	214.7 ± 126.1	(61.4–432.8)	106.7 ± 73.5	(46.2–272.3)	n.s.

Data were obtained using the gating and analysis strategies described in Figure 1, where representative dot plots of these two conventional NK-cell subsets are presented.

Results are expressed as mean ± standard deviation (minimum–maximum) of the percentage of cells expressing each of the chemokine receptors analyzed within each CD56^+^ NK-cell population as well as mean ± standard deviation (minimum–maximum) of the mean fluorescence intensity (MFI) and coefficient of variation (CV) of expression.

n.s.: not statistically significant.

**Table 2:** Relative representation and light scatter properties of blood CD56^+low^ CCR5/CXCR3^−^, CD56^+int^ CCR5/CXCR3^+^, and CD56^+high^ CCR5/CXCR3^+^ NK-cell subsets.

	A		B		C		*P* values		
	CD56^+low^ CCR5/CXCR3^−^		CD56^+int^ CCR5/CXCR3^+^		CD56^+high^ CCR5/CXCR3^+^		A versus B	B versus C	A versus C
% CD56^+^ NK-cells	90.3 ± 3.9	(83.4–98.0)	6.1 ± 4.0	(1.2–14.6)	3.7 ± 2.3	(0.9–8.5)	—	—	—
FSC	299 ± 9	(285–315)	303 ± 10	(284–320)	311 ± 11	(291–324)	n.s.	0.044	0.041
tSSC	151 ± 7	(140–163)	156 ± 7	(141–170)	159 ± 5	(147–168)	n.s.	n.s.	n.s.

Data were obtained using the gating and analysis strategies described in Figure 2, where representative dot plots of these three NK-cell subsets are presented.

Results are expressed as mean ± standard deviation (minimum–maximum) of the percentage of each NK-cell subset within total CD56^+^ NK-cells and as mean ± standard deviation (minimum–maximum) of the transformed side scatter (tSSC) and forward scatter (FSC) channel of each NK-cell subset.

n.s.: not statistically significant.

**Table 3:** CD56 and CD16 expression on blood CD56^+low^ CCR5/CXCR3^−^, CD56^+int^ CCR5/CXCR3^+^, and CD56^+high^ CCR5/CXCR3^+^ NK-cell subsets.

		A		B		C		*P* values		
		CD56^+low^ CCR5/CXCR3^−^		CD56^+int^ CCR5/CXCR3^+^		CD56^+high^ CCR5/CXCR3^+^		A versus B	B versus C	A versus C
CD56	% (+) cells	100.0 ± 0.0	(100.0-100.0)	100.0 ± 0.0	(100.0-100.0)	100.0 ± 0.0	(100.0-100.0)	—	—	—
	MFI	466 ± 108	(303–623)	615 ± 149	(416–999)	2926 ± 578	(2161–3993)	0.022	<0.001	<0.001
	CV	57 ± 7	(41–66)	72 ± 11	(53–93)	37 ± 5	(27–48)	0.001	<0.001	<0.001

CD16	% (+) cells	99.9 ± 0.1	(99.9–100.0)	64.6 ± 23.6	(26.8–96.2)	28.7 ± 9.9	(9.9–43.5)	<0.001	<0.001	<0.001
	MFI	226 ± 107	(55–372)	84 ± 55	(25–201)	47 ± 18	(21–47)	0.001	0.026	<0.001
	CV	45 ± 5	(38–55)	79 ± 21	(45–122)	81 ± 21	(44–118)	<0.001	n.s.	<0.001

Data were obtained using the gating and analysis strategies described in Figure 2, where representative dot plots of these three NK-cell subsets are presented.

Results are expressed as mean ± standard deviation (minimum–maximum) of the percentage of positive (+) cells within each CD56^+^ NK-cell population and of the mean fluorescence intensity (MFI) and coefficient of variation (CV) of CD16 and CD56 expression.

n.s.: not statistically significant.

**Table 4:** CD57, CD62L, CD94, CD122, and CD158a expression on peripheral blood CD56^+low^ CCR5/CXCR3^−^, CD56^+int^ CCR5/CXCR3^+^, and CD56^+high^ CCR5/CXCR3^+^ NK-cell subsets.

		A		B		C		*P* values		
		CD56^+low^ CCR5/CXCR3^−^		CD56^+int^ CCR5/CXCR3^+^		CD56^+high^ CCR5/CXCR3^+^		A versus B	B versus C	A versus C
CD57	% (+) cells	66.3 ± 15.6	(36.7–87.4)	15.3 ± 13.7	(1.7–42.7)	1.3 ± 1.4	(0.0–4.3)	<0.001	<0.001	<0.001
	MFI	700 ± 436	(217–1557)	355 ± 166	(150–703)	165 ± 219	(9–574)	0.018	0.015	<0.001
	CV	114 ± 34	(77–194)	130 ± 35	(80–205)	85 ± 54	(6–155)	n.s.	n.s.	n.s.

CD62L	% (+) cells	35.4 ± 20.4	(9.6–73.5)	77.3 ± 19.0	(41.9–95.7)	97.3 ± 2.4	(92.8–100.0)	<0.001	<0.001	<0.001
	MFI	49 ± 9	(38–65)	119 ± 21	(77–147)	139 ± 30	(91–172)	<0.001	0.057	<0.001
	CV	83 ± 11	(72–109)	56 ± 7	(46–71)	47 ± 9	(35–69)	<0.001	0.010	<0.001

CD94	% (+) cells	47.8 ± 13.7	(34.1–74.1)	91.4 ± 6.0	(79.2–98.4)	98.3 ± 1.5	(94.5–100.0)	<0.001	<0.001	<0.001
	MFI	71 ± 18	(50–106)	129 ± 34	(77–211)	228 ± 34	(175–286)	<0.001	<0.001	<0.001
	CV	56 ± 7	(44–69)	56 ± 11	(41–85)	47 ± 8	(31–56)	n.s.	0.039	0.039

CD122	% (+) cells	100.0 ± 0.0	(100.0-100.0)	100.0 ± 0.0	(100.0-100.0)	100.0 ± 0.0	(100.0-100.0)	n.s.	n.s.	n.s.
	MFI	46 ± 11	(31–63)	77 ± 20	(49–107)	117 ± 32	(79–182)	<0.001	0.001	<0.001
	CV	50 ± 12	(35–72)	56 ± 9	(43–79)	46 ± 5	(34–54)	n.s.	<0.001	<0.001

CD158a	% (+) cells	38.9 ± 30.0	(7.9–92.9)	9.9 ± 9.0	(0.7–30.0)	4.1 ± 4.9	(0.1–19.3)	0.001	0.030	<0.001
	MFI	30 ± 8	(18–42)	35 ± 11	(22–57)	41 ± 13	(20–68)	n.s.	n.s.	n.s.
	CV	61 ± 21	(27–98)	63 ± 20	(31–96)	69 ± 29	(35–126)	n.s.	n.s.	n.s.

Data were obtained using the gating and analysis strategies described in Figure 2, where representative dot plots of these three NK-cell subsets are presented.

Results are expressed as mean ± standard deviation (minimum–maximum) of the percentage of positive (+) cells within each CD56^+^ NK-cell population, and as the mean fluorescence intensity (MFI) and coefficient of variation (CV) of CD57, CD62L, CD94, CD122, and CD158a expression in cells that stained positively for these antigens.

n.s.: not statistically significant.

**Table 5:** Homeostatic and inflammatory chemokines (CK) and chemokine receptors (CKR) involved in cell mediated immune responses and their relevance for colocalization of the NK-cell subsets with other cells of the innate-dendritic cells (DC), monocytes, neutrophils, and adaptive (T cells) immune system. Colocalization of CD56^+high^, CD56^+int^, and CD56^+high^ NK-cells with other immune cells is signaled in blue, green, and red, respectively. Black squares inside the cells indicate positive interactions of the CK and the CKR as well as the CKR expressing cells. CD56^+int^ NK-cells are transitional NK-cells, whose properties are intermediate between those of CD56^+high^ and CD56^+low^ NK-cells. CD56^+high^ and CD56^+low^ NK-cells are both able to produce cytokines and chemokines, upon stimulation with monokines and target cell recognition, respectively. The CK produced by NK-cells (e.g., MIP-1*α*, MIP-1*β*, and RANTES) not only attracted proinflammatory monocytes (via CCR1 and CCR5), immature myeloid DC (via CCR1 and CCR5), Th1 cells, and CTL (via CCR5) but also activated neutrophils (via CCR1) to the sites of inflammation. The cytokines produced by the NK-cells (e.g., IFN-*γ*, TNF-*α*) activate monocytes, DC, neutrophils, and Th1/CTL, thereby potentiating the cell mediated immune responses. For instance, activated monocytes/DC are able to produce IFN-*γ* inducible cytokines—CXCL9 (MIG), CXCL10 (IP-10), and CXCL11 (I-TAC), which recruit more CXCR3 expressing cells (CD56^+high^ NK-cells, Th1 cells, and CTL). In addition, CXCL8 (IL-8) produced in inflamed tissues by locally resident activated cells, such as macrophages, epithelial cells, and endothelial cells, recruits neutrophils, CD56^+low^ NK-cells, and CTL, via interaction with CXCL1 and CXCL2.

Chemokine receptors expressed	Chemokines produced																		Chemokine receptor expressing											
	Inflammatory (inducible)															Homeostatic (constitutive)			NK-cells				Other cells involved in cell mediated immune responses							
	CCL-3 (MIP-1*α*)	CCL-4 (MIP-1*β*)	CCL-5 (RANTES)	CCL2 (MCP-1)	CCL8 (MCP-2)	CCL7 (MCP-3)	CCL13 (MCP-4)	CCL20 (LARC)	CXCL10 (IP-10)	CXCL9 (MIG)	CXCL11 (I-TAC)	CCL22 (MDC)	CCL17 (TARC)	CXCL8 (IL-8)	CXCL1 (GRO-*α*)	CCL19 (ELC)	CCL21 (SLC)	CXCL12 (SDF-1)	NK CD56^+high^	NK CD56^+int^		NK CD56^+low^	Immature mDC	Mature myeloid DC	Conventional monocytes (CD16^−^)	Proinflammatory monocytes (CD16^+^)	Naive T cells	Th1 T cells	Cytotoxic T Lymphocytes	Neutrophils
CCR1	■		■		■	■																	■		■	■		□		□
CCR2				■	■	■	■																■		■			■		
CCR5	■	■	■																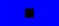	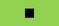			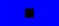			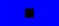		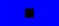	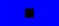	
CCR6								■															□							
CXCR1														■							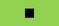	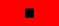	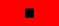						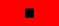	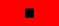
CXCR2														■	■						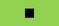	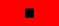								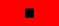
CXCR3									■	■	■								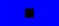	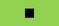								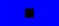	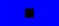	

CCR4												■	■											■						
CCR7																■	■		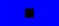					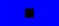			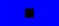			
CXCR4																		■	■	■		■		■	■	■	■	□		

## Abbreviations

ADCC: Antibody dependent cell cytotoxicity
APC: Allophycocyanin
BC: Beckman Coulter
BD: Becton Dickinson
BDB: Becton Dickinson Biosciences
BM: Bone marrow
CK: Chemokines
CKR: Chemokine receptors
CTL: Cytotoxic T lymphocytes
DC: Dendritic cells
ELR: Glutamic acid-leucine-arginine tripeptide motif
Fc*γ*RIIIa: Low affinity receptor for IgG Fc and alpha chain (CD16)
FCRL: Fc receptor-like
FITC: Fluorescein isothiocyanate
FSC: Forward light scatter
IFN-*γ*: Interferon-gamma
IL: Interleukin
ILT: Immunoglobulin-like transcript
IP-10: Interferon-induced protein of 10 kD (CXCL10)
I-TAC: Interferon-inducible T cell alpha chemoattractant (CXCL11)
KIR: Killer cell immunoglobulin-like receptors
KLR: Killer cell lectin type receptors
LARC: Liver- and activation-regulated chemokine (CCL20)
LN: Lymph nodes
MCP-2: Monocyte chemotactic protein-2 (CCL8)
MDC: Macrophage-derived chemokine (CCL22)
MHC: Major Histocompatibility Complex
MIG: Monokine induced by gamma-interferon (CXCL9)
MIP-1*α*: Macrophage inflammatory protein 1 alpha (CCL3)
MIP-1*β*: Macrophage inflammatory protein 1 beta (CCL4)
NCAM: Neural cell adhesion molecule (CD56)
NCR: Natural cytotoxicity receptors
NK: Natural killer
PB: Peripheral blood
PC5: PE-Cyanine 5
PE: Phycoerythrin
PH: Pharmingen
RANTES: Regulated upon activation, normal T cell expressed and secreted (CCL5)
SSC: Side light scatter
SDF-1: Stromal cell derived factor type 1 (CXCL12)
TARC: Thymus and activation-regulated chemokine (CCL17)
TGF-*β*: Transformed growth factor beta
Th: T helper
TNF-*α*: Tumor necrosis factor alpha.

## References

[1] Vivier E., Tomasello E., Baratin M., Walzer T., Ugolini S. (2008). Functions of natural killer cells. *Nature Immunology*.

[2] Degli-Esposti M. A., Smyth M. J. (2005). Close encounters of different kinds: dendritic cells and NK cells take centre stage. *Nature Reviews Immunology*.

[3] Brilot F., Strowig T., Munz C. (2008). NK cells interactions with dendritic cells shape innate and adaptive immunity. *Frontiers in Bioscience*.

[4] Moretta A., Marcenaro E., Parolini S., Ferlazzo G., Moretta L. (2008). NK cells at the interface between innate and adaptive immunity. *Cell Death and Differentiation*.

[5] Crouse J., Xu H. C., Lang P. A., Oxenius A. (2015). NK cells regulating T cell responses: mechanisms and outcome. *Trends in Immunology*.

[6] Costantini C., Cassatella M. A. (2011). The defensive alliance between neutrophils and NK cells as a novel arm of innate immunity. *Journal of Leukocyte Biology*.

[7] Scapini P., Cassatella M. A. (2014). Social networking of human neutrophils within the immune system. *Blood*.

[8] Moretta A., Bottino C., Vitale M. (2001). Activating receptors and coreceptors involved in human natural killer cell-mediated cytolysis. *Annual Review of Immunology*.

[9] Borrego F., Kabat J., Kim D.-K. (2002). Structure and function of major histocompatibility complex (MHC) class I specific receptors expressed on human natural killer (NK) cells. *Molecular Immunology*.

[10] Natarajan K., Dimasi N., Wang J., Mariuzza R. A., Margulies D. H. (2002). Structure and function of natural killer cell receptors: multiple molecular solutions to self, nonself discrimination. *Annual Review of Immunology*.

[11] Maghazachi A. A. (2010). Role of chemokines in the biology of natural killer cells. *Current Topics in Microbiology and Immunology*.

[12] Paolini R., Bernardini G., Molfetta R., Santoni A. (2014). NK cells and interferons. *Cytokine Growth Factor Reviews*.

[13] Möller M. J., Kammerer R., von Kleist S. (1998). A distinct distribution of natural killer cell subgroups in human tissues and blood. *International Journal of Cancer*.

[14] Morris M., Ley K. (2004). Trafficking of natural killer cells. *Current Molecular Medicine*.

[15] Carrega P., Ferlazzo G. (2012). Natural killer cell distribution and trafficking in human tissues. *Frontiers in Immunology*.

[16] Bernardini G., Santoni A. (2014). The pathophysiological role of chemokines in the regulation of NK cell tissue homing. *Critical Reviews in Oncogenesis*.

[17] Sharma R., Das A. (2014). Organ-specific phenotypic and functional features of NK cells in humans. *Immunology Research*.

[18] Sojka D. K., Tian Z., Yokoyama W. M. (2014). Tissue-resident natural killer cells and their potential diversity. *Seminars in Immunology*.

[19] Santoni A., Carlino C., Gismondi A. (2008). Uterine NK cell development, migration and function. *Reproductive BioMedicine Online*.

[20] Moffett A., Colucci F. (2014). Uterine NK cells: active regulators at the maternal-fetal interface. *Journal of Clinical Investigation*.

[21] de Bruin A. M., Voermans C., Nolte M. A. (2014). Impact of interferon-*γ* on hematopoiesis. *Blood*.

[22] Diederich M., Morceau F., Diederich M. (2009). Pro-inflammatory cytokine-mediated anemia: Regarding molecular mechanisms of erythropoiesis. *Mediators of Inflammation*.

[23] Cooper M. A., Fehniger T. A., Caligiuri M. A. (2001). The biology of human natural killer-cell subsets. *Trends in Immunology*.

[24] Poli A., Michel T., Thérésine M., Andrès E., Hentges F., Zimmer J. (2009). CD56^bright^ natural killer (NK) cells: an important NK cell subset. *Immunology*.

[25] Baume D. M., Robertson M. J., Levine H., Manley T. J., Schow P. W., Ritz J. (1992). Differential responses to interleukin 2 define functionally distinct subsets of human natural killer cells. *European Journal of Immunology*.

[26] Carson W. E., Fehniger T. A., Caligiuri M. A. (1997). CD56^bright^ natural killer cell subsets: characterization of distinct functional responses to interleukin-2 and the c-kit ligand. *European Journal of Immunology*.

[27] Frey M., Packianathan N. B., Fehniger T. A. (1998). Differential expression and function of L-selectin on CD56^bright^ and CD56^dim^ natural killer cell subsets. *The Journal of Immunology*.

[28] Jacobs R., Hintzen G., Kemper A. (2001). CD5656^bright^ cells differ in their KIR repertoire and cytotoxic features from CD56^dim^ NK cells. *European Journal of Immunology*.

[29] Lima M., Dos Anjos Teixeira M., Queirós M. L. (2001). Immunophenotypic characterization of normal blood CD56^lo+^ versus CD56^hi+^ NK-cell subsets and its impact on the understanding of their tissue distribution and functional properties. *Blood Cells, Molecules, and Diseases*.

[30] Aguado E., Santamaría M., Gallego M. D., Peña J., Molina I. J. (1999). Functional expression of CD43 on human natural killer cells. *Journal of Leukocyte Biology*.

[31] André P., Spertini O., Guia S. (2000). Modification of P–selectin glycoprotein ligand–1 with a natural killer cell–restricted sulfated lactosamine creates an alternate ligand for L–selectin. *Proceedings of the National Academy of Sciences of the United States of America*.

[32] Nagler A., Lanier L. L., Cwirla S., Phillips J. H. (1989). Comparative studies of human FcRIII-positive and negative natural killer cells. *Journal of Immunology*.

[33] Cooper M. A., Fehniger T. A., Turner S. C. (2001). Human natural killer cells: a unique innate immunoregulatory role for the CD56^bright^ subset. *Blood*.

[34] Fauriat C., Long E. O., Ljunggren H.-G., Bryceson Y. T. (2010). Regulation of human NK-cell cytokine and chemokine production by target cell recognition. *Blood*.

[35] Parrish-Novak J., Dillon S. R., Nelson A. (2000). Interleukin 21 and its receptor are involved in NK cell expansion and regulation of lymphocyte function. *Nature*.

[36] Sivori S., Cantoni C., Parolini S. (2003). IL-21 induces both rapid maturation of human CD34^+^ cell precursors towards NK cells and acquisition of surface killer Ig-like receptors. *European Journal of Immunology*.

[37] Loza M. J., Perussia B. (2004). The IL–12 signature: NK–cell terminal CD56^+high^ stage and effector functions. *Journal of Immunology*.

[38] Ferlazzo G., Thomas D., Lin S.-L. (2004). The abundant NK cells in human secondary lymphoid tissues require activation to express killer cell Ig-like receptors and become cytolytic. *Journal of Immunology*.

[39] Romagnani C., Juelke K., Falco M. (2007). CD56^bright^CD16-killer Ig-like receptor-NK cells display longer telomeres and acquire features of CD56dim NK cells upon activation. *Journal of Immunology*.

[40] Chan A., Hong D.-L., Atzberger A. (2007). CD56^bright^ human NK cells differentiate into CD56^dim^ cells: role of contact with peripheral fibroblasts. *The Journal of Immunology*.

[41] Ouyang Q., Baerlocher G., Vulto I., Lansdorp P. M. (2007). Telomere length in human natural killer cell subsets. *Annals of the New York Academy of Sciences*.

[42] Dulphy N., Haas P., Busson M. (2008). An unusual CD56^bright^CD16^low^ NK cell subset dominates the early posttransplant period following HLA-matched hematopoietic stem cell transplantation. *Journal of Immunology*.

[43] Campbell D. J., Kim C. H., Butcher E. C. (2003). Chemokines in the systemic organization of immunity. *Immunological Reviews*.

[44] International Union of Immunological Societies/World Health Organization (IUIS/WHO) Subcommittee on Chemokine Nomenclature (2003). Chemokine/chemokine receptor nomenclature. *Cytokine*.

[45] Campbell J. J., Qin S., Unutmaz D. (2001). Unique subpopulations of CD56^+^ NK and NK-T peripheral blood lymphocytes identified by chemokine receptor expression repertoire. *Journal of Immunology*.

[46] Berahovich R. D., Lai N. L., Wei Z., Lanier L. L., Schall T. J. (2006). Evidence for NK–cell subsets based on chemokine receptor expression. *Journal of Immunology*.

[47] Fehniger T. A., Cooper M. A., Nuovo G. J. (2003). CD56^bright^ natural killer cells are present in human lymph nodes and are activated by T cell-derived IL-2: a potential new link between adaptive and innate immunity. *Blood*.

[48] Vitale M., Della Chiesa M., Carlomagno S. (2004). The small subset of CD56^bright^CD16^−^ natural killer cells is selectively responsible for both cell proliferation and interferon-gamma production upon interaction with dendritic cells. *European Journal of Immunology*.

[49] Romagnani P., Lasagni L., Annunziato F., Serio M., Romagnani S. (2004). CXC chemokines: the regulatory link between inflammation and angiogenesis. *Trends in Immunology*.

[50] Groom J. R., Luster A. D. (2011). CXCR3 ligands: redundant, collaborative and antagonistic functions. *Immunology & Cell Biology*.

[51] Cole K. E., Strick C. A., Paradis T. J. (1998). Interferon-inducible T cell alpha chemoattractant (I-TAC): a novel non- ELR CXC chemokine with potent activity on activated T cells through selective high affinity binding to CXCR3. *Journal of Experimental Medicine*.

[52] Loetscher M., Loetscher P., Brass N., Meese E., Moser B. (1998). Lymphocyte-specific chemokine receptor CXCR3: regulation, chemokine binding and gene localization. *European Journal of Immunology*.

[53] Combadiere C., Ahuja S. K., Tiffany H. L., Murphy P. M. (1996). Cloning and functional expression of CC CKR5, a human monocytg CC chemokine receptor selective for MIP-1*α*, MIP-1*β*, and RANTES. *Journal of Leukocyte Biology*.

[54] Raport C. J., Gosling J., Schweickart V. L., Gray P. W., Charo I. F. (1996). Molecular cloning and functional characterization of a novel human CC chemokine receptor (CCR5) for RANTES, MIP-1*β*, and MIP-1*α*. *The Journal of Biological Chemistry*.

[55] Bleul C. C., Farzan M., Choe H. (1996). The lymphocyte chemoattractant SDF-1 is a ligand for LESTR/fusin and blocks HIV-1 entry. *Nature*.

[56] Oberlin E., Amara A., Bachelerie F. (1996). The CXC chemokine SDF-1 is the ligand for LESTR/fusin and prevents infection by T-cell-line-adapted HIV-1. *Nature*.

[57] Juarez J., Bendall L. (2004). SDF-1 and CXCR4 in normal and malignant hematopoiesis. *Histology and Histopathology*.

[58] Bonecchi R., Bianchi G., Bordignon P. P. (1998). Differential expression of chemokine receptors and chemotactic responsiveness of type 1 T helper cells (Th1s) and Th2s. *Journal of Experimental Medicine*.

[59] Campbell J. J., Haraldsen G., Pan J. (1999). The chemokine receptor CCR4 in vascular recognition by cutaneous but not intestinal memory T cells. *Nature*.

[60] Imai T., Baba M., Nishimura M., Kakizaki M., Takagi S., Yoshie O. (1997). The T cell-directed CC chemokine TARC is a highly specific biological ligand for CC chemokine receptor 4. *The Journal of Biological Chemistry*.

[61] Imai T., Chantry D., Raport C. J. (1998). Macrophage-derived chemokine is a functional ligand for the CC chemokine receptor 4. *The Journal of Biological Chemistry*.

[62] Liao F., Rabin R. L., Smith C. S., Sharma G., Nutman T. B., Farber J. M. (1999). CC-chemokine receptor 6 is expressed on diverse memory subsets of T cells and determines responsiveness to macrophage inflammatory protein 3*α*. *Journal of Immunology*.

[63] Schutyser E., Struyf S., van Damme J. (2003). The CC chemokine CCL20 and its receptor CCR6. *Cytokine & Growth Factor Reviews*.

[64] Charbonnier A.-S., Kohrgruber N., Kriehuber E., Stingl G., Rot A., Maurer D. (1999). Macrophage inflammatory protein 3*α* is involved in the constitutive trafficking of epidermal langerhans cells. *The Journal of Experimental Medicine*.

[65] Qin S., Rottman J. B., Myers P. (1998). The chemokine receptors CXCR3 and CCR5 mark subsets of T cells associated with certain inflammatory reactions. *Journal of Clinical Investigation*.

[66] Morohashi H., Miyawaki T., Nomura H. (1995). Expression of both types of human interleukin–8 receptors on mature neutrophils, monocytes, and natural killer cells. *Journal of Leukocyte Biology*.

[67] Murphy P. M. (1997). Neutrophil receptors for interleukin-8 and related CXC chemokines. *Seminars in Hematology*.

[68] Weber C., Belge K.-U., Von Hundelshausen P. (2000). Differential chemokine receptor expression and function in human monocyte subpopulations. *Journal of Leukocyte Biology*.

[69] Pridgeon C., Lennon G. P., Pazmany L., Thompson R. N., Christmas S. E., Moots R. J. (2003). Natural killer cells in the synovial fluid of rheumatoid arthritis patients exhibit a CD56^bright^, CD94^bright^, CD158^negative^ phenotype. *Rheumatology*.

[70] Ottaviani C., Nasorri F., Bedini C., de Pità O., Girolomoni G., Cavani A. (2006). CD56^bright^CD16^−^ NK cells accumulate in psoriatic skin in response to CXCL10 and CCL5 and exacerbate skin inflammation. *European Journal of Immunology*.

[71] Katchar K., Söderström K., Wahlstrom J., Eklund A., Grunewald J. (2005). Characterisation of natural killer cells and CD56^+^ T-cells in sarcoidosis patients. *European Respiratory Journal*.

[72] Obara H., Nagasaki K., Hsieh C. L. (2005). IFN-gamma, produced by NK cells that infiltrate liver allografts early after transplantation, links the innate and adaptive immune responses. *American Journal of Transplantation*.

[73] Batoni G., Esin S., Favilli F. (2005). Human CD56^bright^ and CD56^dim^ natural killer cell subsets respond differentially to direct stimulation with *Mycobacterium bovis* bacillus Calmette-Guérin. *Scandinavian Journal of Immunology*.

[74] Wang J., Holmes T. H., Cheung R., Greenberg H. B., He X.-S. (2004). Expression of chemokine receptors on intrahepatic and peripheral lymphocytes in chronic hepatitis C infection: its relationship to liver inflammation. *Journal of Infectious Diseases*.

[75] Carrega P., Morandi B., Costa R. (2008). Natural killer cells infiltrating human nonsmall-cell lung cancer are enriched in CD56^bright^CD16^−^ cells and display an impaired capability to kill tumor cells. *Cancer*.

[76] Dalbeth N., Gundle R., Davies R. J. O., Lee Y. C. G., McMichael A. J., Callan M. F. C. (2004). CD56^bright^ NK cells are enriched at inflammatory sites and can engage with monocytes in a reciprocal program of activation. *The Journal of Immunology*.

[77] Nishikawa K., Saito S., Morii T. (1991). Accumulation of CD16^−^CD56^+^ natural killer cells with high affinity interleukin 2 receptors in human early pregnancy decidua. *International Immunology*.

[78] Koopman L. A., Kopcow H. D., Rybalov B. (2003). Human decidual natural killer cells are a unique NK–cell subset with immunomodulatory potential. *Journal of Experimental Medicine*.

[79] Yagel S. (2009). The developmental role of natural killer cells at the fetal-maternal interface. *The American Journal of Obstetrics and Gynecology*.

[80] Takata H., Tomiyama H., Fujiwara M., Kobayashi N., Takiguchi M. (2004). Cutting edge: expression of chemokine receptor CXCR1 on human effector CD8^+^ T cells. *The Journal of Immunology*.

[81] Gomita K., Sato K., Yoshida M., Hagiwara N. (2012). PSGL-1-expressing CD4 T cells induce endothelial cell apoptosis in perimenopausal women. *Journal of Atherosclerosis and Thrombosis*.

[82] Bhatnagar N., Hong H. S., Krishnaswamy J. K. (2010). Cytokine-activated NK cells inhibit PMN apoptosis and preserve their functional capacity. *Blood*.

[83] Costantini C., Micheletti A., Calzetti F., Perbellini O., Pizzolo G., Cassatella M. A. (2010). Neutrophil activation and survival are modulated by interaction with NK cells. *International Immunology*.

[84] Costantini C., Micheletti A., Calzetti F. (2011). On the potential involvement of CD11d in co-stimulating the production of interferon-*γ* by natural killer cells upon interaction with neutrophils via intercellular adhesion molecule-3. *Haematologica*.

[85] Hudspeth K., Pontarini E., Tentorio P. (2013). The role of natural killer cells in autoimmune liver disease: a comprehensive review. *Journal of Autoimmunity*.

[86] Zimmermann H. W., Seidler S., Gassler N. (2011). Interleukin-8 is activated in patients with chronic liver diseases and associated with hepatic macrophage accumulation in human liver fibrosis. *PLoS ONE*.

[87] Inngjerdingen M., Damaj B., Maghazachi A. A. (2001). Expression and regulation of chemokine receptors in human natural killer cells. *Blood*.

[88] Kunkel E. J., Campbell J. J., Haraldsen G. (2000). Lymphocyte CC chemokine receptor 9 and epithelial thymus-expressed chemokine (TECK) expression distinguish the small intestinal immune compartment: epithelial expression of tissue-specific chemokines as an organizing principle in regional immunity. *Journal of Experimental Medicine*.

[89] Kunkel E. J., Campbell D. J., Butcher E. C. (2003). Chemokines in lymphocyte trafficking and intestinal immunity. *Microcirculation*.

[90] Yu J., Mao H. C., Wei M. (2010). CD94 surface density identifies a functional intermediary between the CD56^bright^ and CD56^dim^ human NK-cell subsets. *Blood*.

[91] Juelke K., Killig M., Luetke-Eversloh M. (2010). CD62L expression identifies a unique subset of polyfunctional CD56^dim^ NK cells. *Blood*.

[92] Béziat V., Descours B., Parizot C., Debré P., Vieillard V. (2010). NK cell terminal differentiation: correlated stepwise decrease of NKG2A and acquisition of KIRs. *PLoS ONE*.

[93] Lima M., Almeida J., dos Anjos Teixeira M., Queirós M. L., Justiça B., Orfão A. (2002). The ‘ex vivo’ patterns of CD2/CD7, CD57/CD11c, CD38/CD11b, CD45RA/CD45RO, and CD11a/HLA-DR expression identify acute/early and chronic/late NK-cell activation states. *Blood Cells, Molecules, and Diseases*.

[94] Lima M., Almeida J., Teixeira M. A. (2004). Reactive phenotypes after acute and chronic NK-cell activation. *Journal of Biological Regulators & Homeostatic Agents*.

[95] Villamor N., Morice W. G., Chan W. C., Foucar K. K., Swerdlow S. H., Campo E., Harris N. L. (2008). Chronic Iymphoproliferative disorders of NK cells. *WHO Classification of Tumours of Haematopoietic and Lymphoid Tissues*.

[96] Lima M., Almeida J., Montero A. G. (2004). Clinicobiological, immunophenotypic, and molecular characteristics of monoclonal CD56^-/+dim^ chronic natural killer cell large granular lymphocytosis. *American Journal of Pathology*.

[97] Lima M., Spínola A., Fonseca S. (2015). Aggressive mature natural killer cell neoplasms: report on a series of 12 European patients with emphasis on flow cytometry based immunophenotype and DNA content of neoplastic natural killer cells. *Leukemia & Lymphoma*.

